# The Protective Role of Resveratrol against Arsenic Trioxide-Induced Cardiotoxicity

**DOI:** 10.1155/2013/407839

**Published:** 2013-11-14

**Authors:** Weiqian Zhang, Changming Guo, Ruifeng Gao, Ming Ge, Yanzhu Zhu, Zhigang Zhang

**Affiliations:** ^1^College of Veterinary Medicine, Northeast Agricultural University, Harbin 150030, China; ^2^College of Animal Science and Veterinary, Medicine, Jilin University, Changchun 130062, China; ^3^Institute of Special Economic Animal and Plant Science, Chinese Academy of Agricultural Sciences, Jilin 132109, China

## Abstract

Arsenic trioxide (As_2_O_3_) shows substantial anticancer activity in patients with acute promyelocytic leukemia (APL). Unfortunately, limiting the application of this effective agent to APL patients is severe cardiotoxicity. Resveratrol, the natural food-derived polyphenolic compound, is well known for its antioxidant properties and protects the cardiovascular system. But the potential role of resveratrol against As_2_O_3_ in heart via nuclear factor erythroid 2-related factor 2 (Nrf2) and heme oxygenase-1 (HO-1) is unclear. The present study evaluated the effects of pretreatment with resveratrol and As_2_O_3_ on oxidative stress and cardiac dysfunction in rat. In the present study, resveratrol decreased As_2_O_3_-induced reactive oxygen species generation, oxidative DNA damage, and pathological alterations. In addition, cardiac dysfunction parameters, intracellular calcium and arsenic accumulation, glutathione redox ratio, and cAMP deficiency levels were observed in As_2_O_3_-treated rats; these changes were attenuated by resveratrol. Furthermore, resveratrol significantly prohibited the downregulation of both Nrf2 and HO-1 gene expressions that were downregulated by As_2_O_3_, whereas resveratrol did not alter As_2_O_3_-induced nitric oxide formation. Thus, the protective role of resveratrol against As_2_O_3_-induced cardiotoxicity is implemented by the maintenance of redox homeostasis (Nrf2-HO-1 pathway) and facilitating arsenic efflux. Our findings suggest coadministration with resveratrol, and As_2_O_3_ might provide a novel therapeutic strategy for APL.

## 1. Introduction

High concentration of dietary exposure to arsenic and arsenic compounds is considered to increase the risk of human carcinogenesis [1]. However, arsenic has attracted worldwide interest because it shows substantial anticancer activity in individuals with acute promyelocytic leukemia (APL). Unfortunately, the use of these drugs is associated with cardiotoxicity (including a prolonged QT interval and prolonged action potential), *torsades de pointes*, and sudden cardiac death [2–5]. This may involve multiple mechanisms, including the generation of reactive oxygen species (ROS) in cardiomyocytes, oxidative DNA damage, and arsenic accumulation, [6–8]. However, proper drug that has the protective ability of the heart to protect against arsenic toxicity in the clinical practice is insufficient. 

Resveratrol (3,5,4′-trihydroxy-trans-stilbene) is a plant-derived polyphenolic compound belonging to a class of stilbenes, found abundantly in certain grapes, roots, berries, and peanuts. Resveratrol has been shown to exert various cardiovascular protective effects in myocardial ischemic-reperfusion injury and atherosclerosis [9, 10], metabolic diseases [11], and in aged mice [12–14]. It was also reported that resveratrol could protect against cardiotoxicity in As_2_O_3_-exposed mouse by the increase in the activities of antioxidant enzymes in the heart and antiapoptotic activity in H9c2 cardiomyocytes [15]. However, whether resveratrol can attenuate the As_2_O_3_-induced cardiotoxicity mediated by improving cardiac function through redox signaling mechanisms and the decrease in arsenic accumulation is yet to be determined. The present study was undertaken to explore this problem.

## 2. Materials and Methods

### 2.1. Animals and Chemicals

All procedures used in this study were approved by the Institutional Animal Care and Use Committee of Northeast Agricultural University. Six-week-old male Wistar rats from the Experimental Animal Centre of Harbin Medical University (Harbin, China) were housed in the Animal Quarters of the Northeast Agricultural University at 22°C on a 12-hour light-dark cycle. They were allowed free access to standard rodent chow and tap water. Thirty-two rats were randomly assigned to four groups: control, As_2_O_3_-treated, As_2_O_3_ + resveratrol, and resveratrol-treated. All treatments were given via the caudal vein on alternate days for 4 days (i.e., days: 1, 3, 5, and 7) with measurements being made on the 8th day. As_2_O_3_ (Harbin Yida Pharmaceutical Co. Ltd., Harbin, China) was administered 3 mg/kg; resveratrol (Sigma-Aldrich, St. Louis, MO, USA) was administered 8 mg/kg; in the As_2_O_3_ + resveratrol group, rats were given resveratrol 1 h prior to As_2_O_3_ administration. Dose selection is based on the literature [14]. An equal amount of 0.9% normal saline was administered as vehicles to control rats. On the 8th day, rats were given ether anesthesia and sacrificed. 

### 2.2. Biochemical Analysis

Blood was collected from puncturing the retro-orbital venous sinus and immediately centrifuged at 8,000 ×g for 10 min at 4°C to separate serum. Serum lactate dehydrogenase (LDH), creatine kinase (CK), creatine kinase MB (CK-MB), and aspartate aminotransferase (AST) were measured using a commercial kit from Jiancheng Bio-engineering Institute (Nanjing, China), following the manufacturer's instructions. 

### 2.3. Measurement of ROS, 8-Hydroxy-2-deoxyguanosine (8-OHdG) and the Ratio Reduced Glutathione (GSH) to Oxidized Glutathione (GSSG)

Cardiac tissues were homogenized in phosphate-buffered saline (pH 7.4) using an Ultrathurax T25 Homogenisator and centrifuged at 10,000 ×g for 10 min at 4°C. ROS production of cardiac tissue was determined by 2′, 7′-dichlorofluorescein diacetate (DCF-DA, Invitrogen) assay, in which highly fluorescent DCF can be converted by cellular peroxides, as previously reported by Maxwell et al. [16]. The DNA of each sample was extracted using a DNeasy tissue kit (QIAGEN, Valencia, CA, USA), and 8-OHdG was measured using an oxidative DNA damage enzyme-linked immunosorbent assay (ELISA) kit (Cell Biolaboratories, San Diego, CA, USA), following the manufacturer's instructions. Supernatant glutathione was determined by the method as described in [17], and the ratio of GSH to GSSG was calculated.

### 2.4. Histological Analysis

Cardiac tissues were quickly removed. For light microscopic (BX-FM; Olympus, Tokyo, Japan) observation, cardiac tissues were fixed by immersion in 10% formaldehyde solution for 24 h at 37°C; then paraffin sections (4 *μ*m) were cut and stained with hematoxylin and eosin. 

### 2.5. Determination of Arsenic Accumulation in the Heart

The arsenic contents in cardiac tissues of all rats were analyzed following the method in the literature [18] with an atomic fluorescence spectrometry system (AFS930; Beijing Jitian Instrument Co. Ltd., Beijing, China). 

### 2.6. Measurements of Cytosolic Free Calcium Ion Ca^2+^ Level

Cardiac cells were digested with 0.25% trypsinase. The cell suspension was washed twice with Tyrode's Solution (in mM: NaCl, 137; KCl, 5.4; MgCl_2_, 1; glucose, 10; HEPES, 10; CaCl_2_, 2; pH 7.4) and loaded with 2 *μ*M Fura-2/AM for 30 min at 37°C in culture medium. The cells were washed three times then incubated for an additional 30 min at 37°C to complete probe de-esterification and resuspended in loading buffer at a density of 10^6^ cells/mL. Fluorescence was monitored with a 970 CRT spectrofluorophotometer at 488 nm for excitation and 530 nm for emission. Maximum and minimum fluorescence values were obtained by adding 0.1% Triton X-100 (plus 5 mM CaCl_2_) and 10 mM EGTA sequentially. Ca^2+^ levels were calculated as previously described [19].

### 2.7. cAMP and Nitric Oxide (NO) Concentration Assay

The supernatant of homogenized cardiac tissue was assayed for cAMP and NO concentration using the enzyme immunoassay kit and the method of chemical colorimetry, respectively, following the manufacturer's instructions (Jiancheng Bio-engineering Institute (Nanjing, China)).

### 2.8. Determination of Nuclear Factor Erythroid 2-Related Factor 2 (Nrf2) and Heme Oxygenase-1 (HO-1) mRNA Level by Quantitative Real-Time PCR Assay

Total RNA was extracted from the cardiac tissue samples using the RNAfast 200 Kit (Fastagen, China). The concentration of total RNA in the extract was quantified spectrophotometrically. RNA integrity was evaluated by the proportion of the ribosomal bands (28S : 18S) after electrophoresis on 1% agarose gel in the presence of ethidium bromide. cDNA was synthesized using 5 *μ*L of total RNA using the reverse transcriptase M-M LV (Promega), as described by the manufacturer's system [20]. Quantitative real-time PCR was carried out using a SYBR Green PCR kit (Bioteke, Peking, China), and PCR amplification was conducted on an ABI PRISM 7500 Sequence Detector System (Perkin-Elmer Applied Biosystems, Foster City, CA, USA). The primer sequences for the genes are as follows: Nrf-2 Forward: 5′-ACT CAT CGA TCC CCT CAC TG-3′, Reverse: 5′-CTA ATG GCA GCA GAG GAA GG-3′; HO-1 Forward: 5′-AAG AGG CTA AGA CCG CCT TC-3′, Reverse: 5′-GCA TAA ATT CCC ACT GCC AC-3′; GAPDH Forward: 5′-GCA TGG CCT TCC GTG TTC C-3′, Reverse: 5′-CTC ATT CTT TGG GAC GTG GTG GG-3′. The expression of mRNA level in each sample was normalized against its GAPDH mRNA level.

### 2.9. Statistical Analysis

Statistical analysis was performed using SPSS ver19.0 (SPSS, Chicago, IL, USA). One-way analysis of variance (Duncan's multiple comparison) was used for the determination of differences in measurements between groups. *P* < 0.05 was considered significant. 

## 3. Results

### 3.1. The Contents of LDH, AST, CK, and CK-MB in Serum

As shown in Table 1, LDH, AST, CK, and CK-MB release from cardiac cells, in the rat treated with As_2_O_3_, were markedly increased compared with those in the control group (*P* < 0.05). Pretreatment of resveratrol resulted in a significant decrease in plasma LDH, AST, CK, and CK-MB release, 20.21%, 37.78%, 59.19%, and 49.88%, respectively, compared with that in the As_2_O_3_-treated group, whereas resveratrol alone did not show significant effect on LDH, AST, CK, and CK-MB activity. 

### 3.2. Effects of Resveratrol on As_**2**_O_**3**_-Induced ROS, 8-OHd, and GSH/GSSG

**Figure 1:**
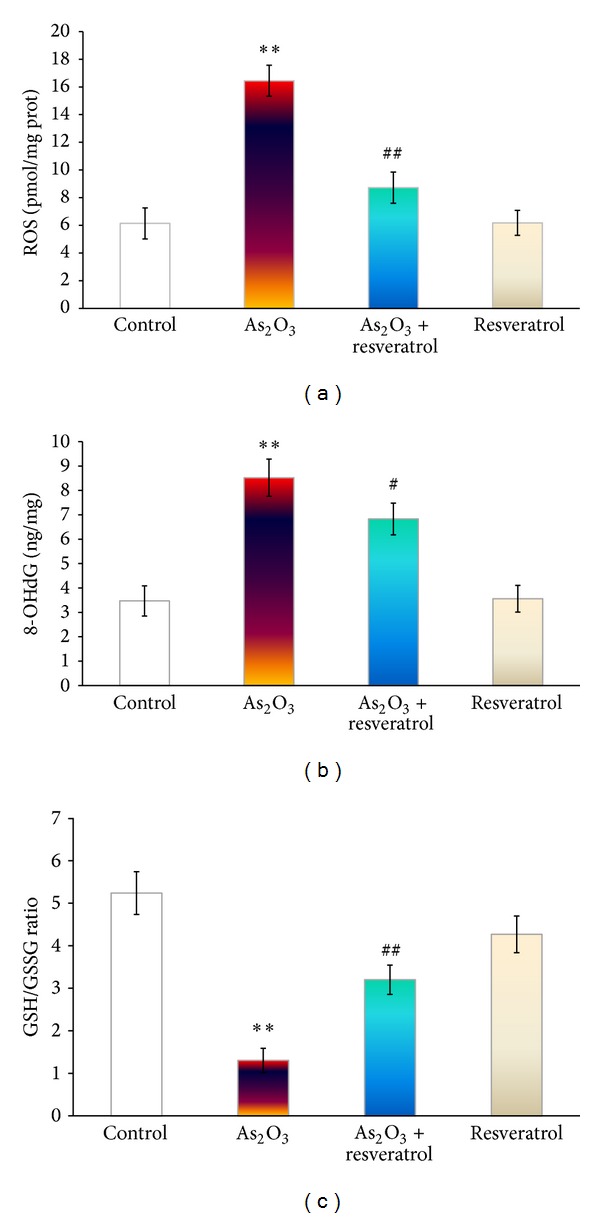
The effect of resveratrol and As_2_O_3_ on ROS (a), 8-OHdG (b), and GSH/GSSG (c) ratio in the heart tissue from control, As_2_O_3_-treated, resveratrol + As_2_O_3_, and resveratrol-treated groups. Values are mean ± S.E. mean; *n* = 8. *P < 0.05 or **P < 0.01 versus control group, ^#^ *P* < 0.05 or ^##^ *P* < 0.01 versus As_2_O_3_-treated group.

After exposure to arsenic for 4 days, the remarkable increase in ROS and 8-OHdG generation was observed in rats' hearts, compared with that in the control group (Figures 1(a) and 1(b)). However, pretreatment with resveratrol partly abolished these changes. In addition, treatment with resveratrol exposure significantly reversed the decrease in As_2_O_3_-induced the ration GSH/GSSG (*P* < 0.01) (Figure 1(c)). 

### 3.3. Effect of Resveratrol on As_**2**_O_**3**_-Induced Cardiomyopathy

**Figure 2:**
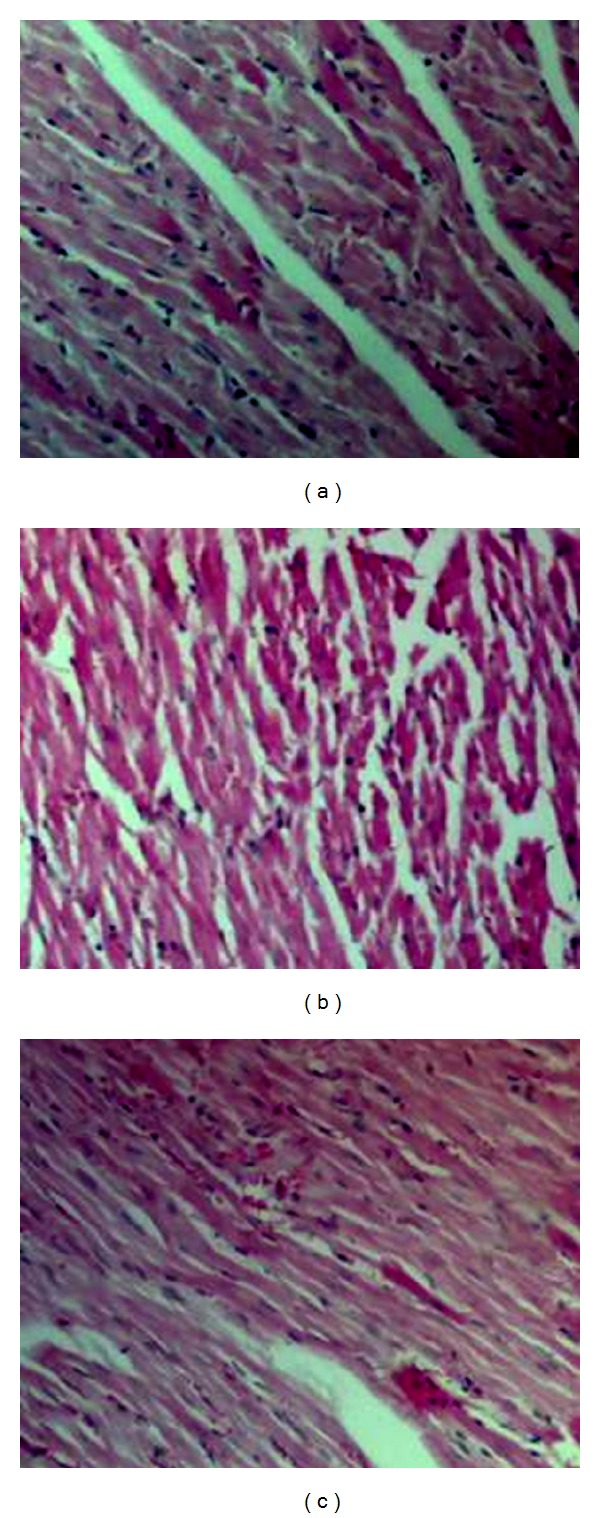
The effect of resveratrol and As_2_O_3_ on cardiac histology. Paraffin sections of heart tissues from control (a), As_2_O_3_-treated (b), and resveratrol + As_2_O_3_ (c) were stained with hematoxylin and eosin (×100 magnification).

Histopathological assessments of different cardiac tissues of rats are shown in Figure 2. Compared with those in the control group, myofibrillar loss and cardiomyocyte necrosis were observed in the hearts of the As_2_O_3_-treated rats (Figure 2(b)). Structural abnormalities in the hearts of As_2_O_3_-treated rats were partly prevented by pretreatment with resveratrol and showed slight myocardial hemorrhage. Resveratrol-treated rats had normal myocardiac morphology (data not shown). 

### 3.4. The Contents of Total Arsenic in the Heart

**Figure 3:**
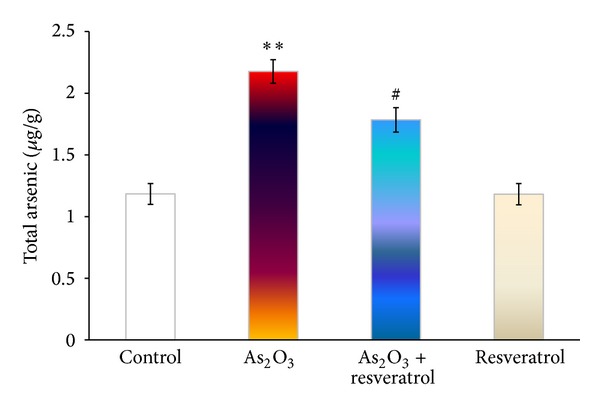
The effect of resveratrol and As_2_O_3_ on total arsenic in the heart. The total arsenic of heart tissues from control, As_2_O_3_-treated, resveratrol + As_2_O_3_, and resveratrol-treated groups was quantified by high-performance liquid chromatography-hydride generation-atomic fluorescence spectrometry. Values are mean ± S.E. mean; *n* = 8. *P < 0.05 or **P < 0.01 versus control group, ^#^ *P* < 0.05 or ^##^ *P* < 0.01 versus As_2_O_3_-treated group.

Figure 3 has shown the contents of total arsenic in the heart. Our results showed that total arsenic content in the heart appeared to be obviously increased compared with As_2_O_3_-treated rats. Pretreatment with resveratrol significantly attenuated arsenic accumulation in the heart compared with that seen in the As_2_O_3_-treated group (*P* < 0.05) (Figure 3).

### 3.5. Effect of Resveratrol on As_**2**_O_**3**_-Induced Intracellular Calcium Accumulation

**Figure 4:**
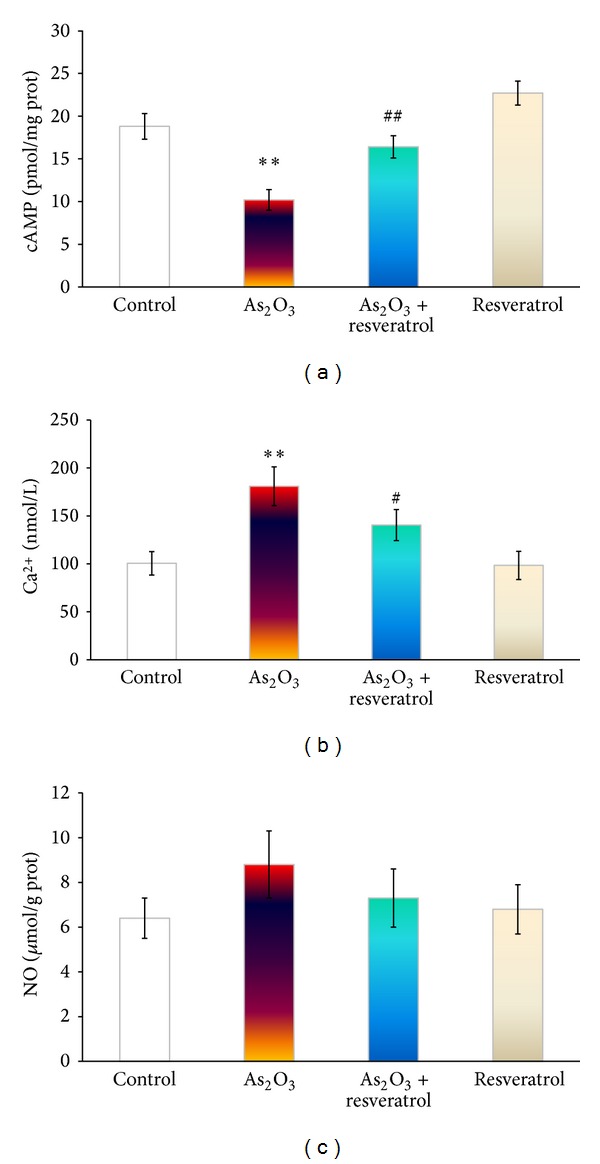
The effect of resveratrol on As_2_O_3_-induced cAMP (a), Ca^2+^ (b), and NO (c) in the heart from control, As_2_O_3_-treated, resveratrol + As_2_O_3_, and resveratrol-treated groups. Values are mean ± S.E. mean; *n* = 8. *P < 0.05 or **P < 0.01 versus control group, ^#^ *P* < 0.05 or ^##^ *P* < 0.01 versus As_2_O_3_-treated group.

The effects of resveratrol on As_2_O_3_-induced intracellular calcium accumulation in the heart are shown in Figure 4(b). Our data indicated Ca^2+^ content was markedly greater in As_2_O_3_-treated group than that in the control group (*P* < 0.01), and resveratrol significantly inhibited this As_2_O_3_-induced that Ca^2+^ accumulation (*P* < 0.05).

### 3.6. Effects of Resveratrol on As_**2**_O_**3**_-Induced cAMP and NO in the Heart

To determine the effect of resveratrol on As_2_O_3_-induced cAMP, concentrations in rats treated with As_2_O_3_ and pretreatment with resveratrol were measured. As expected, treatment of rats with As_2_O_3_ (3 mg/kg i.v.) significantly decreased cAMP concentrations in the heart compared with saline-treated control group, and pretreatment of resveratrol could partly abolish this decrease in cAMP concentration (Figure 4(a)). In contrast, NO concentration in the heart has no statistical change during exposure to As_2_O_3_ and with or without the administration of resveratrol (Figure 4(b)).

### 3.7. Effects of Resveratrol on the mRNA Level of HO-1 and Nrf2

**Figure 5:**
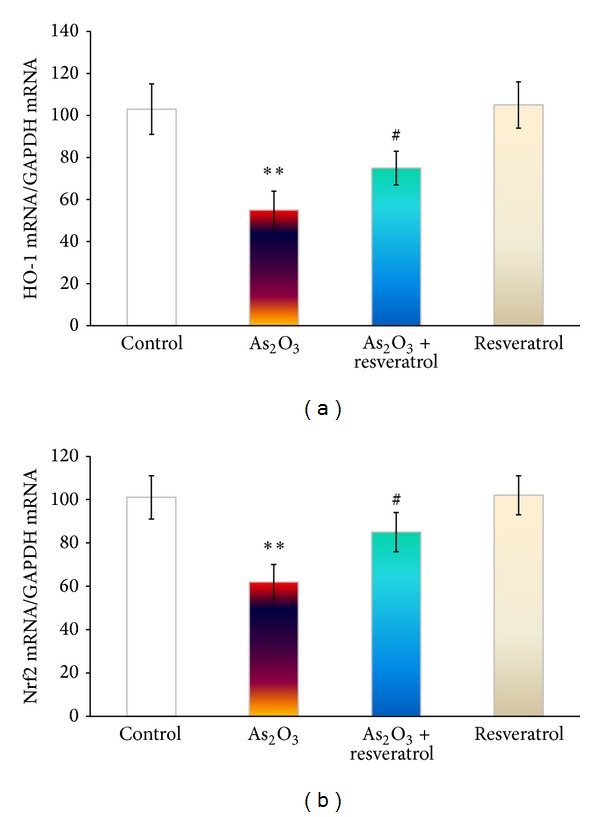
The effect of resveratrol on As_2_O_3_-inducedHO-1 and Nrf2 on the mRNA level in the heart from the control, As_2_O_3_-treated, resveratrol + As_2_O_3_, and resveratrol-treated groups. (a) Real-time quantitative PCR analyses of gene expression levels of HO-1 in cardiac myocytes. (b) Real-time quantitative PCR analyses of gene expression levels of Nrf2 in cardiac myocytes. Values are mean ± S.E. mean; *n* = 8. *P < 0.05 or **P < 0.01 versus control group, ^#^ *P* < 0.05 or ^##^ *P* < 0.01 versus As_2_O_3_-treated group.

To further explore the possible mechanism of As_2_O_3_-induced cardiotoxicity, HO-1 and Nrf2 mRNA with antioxidant and protective properties were selected to determine the effect of resveratrol on cytotoxicity in As_2_O_3_-treated rats. After exposure to As_2_O_3_ on alternate 4 days, both Nrf2 and HO-1 gene expressions in the heart were significantly downregulated, compared with the control group (Figure 5). Treatment with resveratrol significantly prohibited the downregulated Nrf2 and HO-1 gene expressions that were downregulated by As_2_O_3_, compared with As_2_O_3_-treated rats (*P* < 0.01).

## 4. Discussion 

In this study, we investigated cardiac function associated with Nrf2-HO-1 pathway and arsenic accumulation for the protection of resveratrol against As_2_O_3_-induced cardiac injury in Wistar rats *in vivo*. 

Aposhian and Aposhian [21] described that exposure to inorganic arsenic induces cellular oxidative stress through ROS generation. Several studies have indicated that cardiovascular diseases, such as endothelial dysfunction, ischaemia-reperfusion injury, and atherosclerosis, are linked to the release of intracellular ROS [7, 22]. In our studies, LDH, AST, CK, and CK-MB release, which are the most important makers of myocardial injury, disorder and necrosis in response to As_2_O_3_ treatment, were increased, especially CK-MB, which is a more sensitive marker of myocardial injury than total CK activity. In addition, there were various oxidative damages in Wistar rat's heart indicated with the increase of ROS, 8-OHdG formation, and percentage of GSSG/GSH, which resulted in the severe histological alterations, including myofibrillar loss, cardiomyocyte necrosis, and myocardial hemorrhage. Consistent with Ermak and Davies's research on ROS-induced Ca^2+^ dyshomeostasis in the heart [23], our data showed that intracellular calcium accumulation after exposure to As_2_O_3_ is at least partially due to ROS formation induced by As_2_O_3_. 

Under normal circumstances, cells can defend against ROS damage by means of endogenous oxidants, such as glutathione, vitamin C, and vitamin E, as well as with the involvement of various peroxidases in the cellular antioxidant systems. Glutathione redox state correlates with the biological status of the cell [24]. On the other hand, Nrf2 has been demonstrated to be a critical transcription factor that binds to the antioxidant response element in the promoter region of a number of genes, encoding for phase I and phase II antioxidative enzymes and cytoprotective proteins, such as NAD(P)H:quinone acceptor oxidoreductase 1, glutathione S-transferases, the glutamyl cysteine ligase catalytic subunit, and multidrug resistance-associated protein [25]. Hence, Nrf2 pathway is presumably the most important pathway in cells to deal with oxidative stress generated from exposure to exogenous and endogenous chemicals [20]. HO-1 is an enzyme with antioxidant and protective properties during cellular stress [20]. After exposure to As_2_O_3_ (3 mg/kg every alternative day for 4 days), the antioxidant defense system in rats cannot maintain the depletion. Consequently, we observed significantly a decrease in GSH/GSSG ratio and mRNA expression of Nrf2 and HO-1 downregulation (Figure 1). 

There is a considerable interest in the role of constituent in dietary supplement in the prevention and treatment of cardiovascular disease [23]. Furthermore, natural compounds also modulate ROS accounting for the reduction of cell injury in pathological conditions in heart diseases [26]. Unfortunately, several approaches which were able to reduce tissue damage in animal or cell culture models are either not applicable to humans or failed to be beneficial in clinical trials [27, 28]. 

Resveratrol, is an antioxidant found in grapes, red wine, and some other botanical sources with a wide range of biological and pharmacological properties, for example, anti-inflammatory, cardioprotection activity, and anticancer properties [29, 30]. Haskó and Pacher [31] had demonstrated that resveratrol regulated endothelial Nrf2 activation. Therefore, resveratrol administration before As_2_O_3_ treatment diminished As_2_O_3_-induced ROS and 8-OHdG generation mediated by the partly maintenance of GSH/GSSH ratio and mRNA expression of Nrf2 and HO-1 (Figure 5). 

Resveratrol has been shown to regulate cAMP through the competitive inhibition of cAMP-degrading phosphodiesterases, though it has not been clearly demonstrated in myocardial systems [13]. The intracellular cAMP-dependent modulate L-type Ca^2+^ channel has been widely recognized. In cardiac tissue, elevation of Ca^2+^ has been linked to various functional abnormalities, such as ventricular arrhythmia and contractile dysfunction. Also the increase of Ca^2+^ has been suggested to be one of the key signals leading to apoptosis [11, 32]. In our studies, pretreatment with resveratrol attenuated As_2_O_3_-induced calcium overload and cAMP deficiency, suggesting that this might be attributed to the maintenance Ca^2+^ homeostasis by multiple possible ways (Figure 4). Notably, resveratrol administrated had no effect on As_2_O_3_-induced NO overload because of NO dual role in cardiac cells. Taken together, pretreatment with resveratrol ameliorated As_2_O_3_-induced myocardial damage in the heart (Figure 2). We cannot rule out that this result could be due to the improvements in cardiocyte function by heightening their aerobic capacity and autophagy to maintain tissue metabolic homeostasis in the presence of resveratrol [33, 34]. 

**Figure 6:**
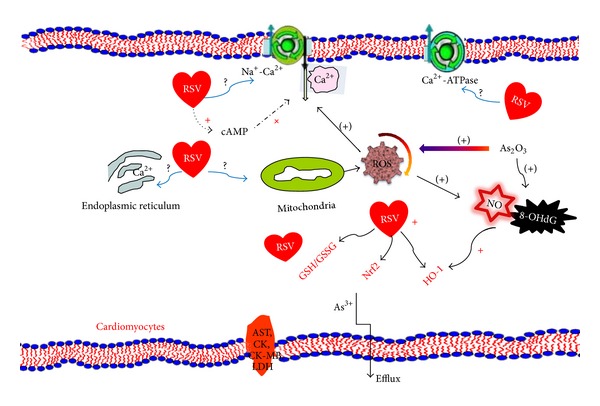
Summary indicating involvement of oxidative stress responses and the possible mechanism associated with Nrf2-HO-1 in As_2_O_3_-induced injury. As_2_O_3_ induces the increase of ROS production from mitochondria in rat cardiac myocytes. ROS triggers Ca^2+^ accumulation, 8-OHdG formation, and GSH deficiency in cardiocytes. RSV scavenges ROS, reduces DNA damage (indicated with 8-OHdG), and preserves GSH and Ca^2+^ homeostasis. Additionally, Nrf2-HO-1, a key signaling pathway involved in cellular oxidative responses, is prohibited by RSV As_2_O_3_-induced downregulation. Taken together, RSV protects the integrity of cardiac myocytes after exposure to As_2_O_3_, thereby decreasing AST, CK, CK-MB, and LDH release, as well as facilitating arsenic efflux. Future studies are required to clarify the mechanism for protecting RSV against As_2_O_3_-induced cardiotoxicity in endoplasmic reticulum and mitochondria. ROS: reactive oxygen species; RSV: resveratrol; + or (+) stands for positive improvement or negative improvement.

Sumi et al. [35] reported that cardiacmyocytes have a weak ability to excrete arsenic into the extracellular space. This sensitivity was attributed to the modest activation of Nrf2, leading to a decrease in the metabolism and excretion of arsenic. It is plausible that resveratrol can facilitate arsenic efflux to reduce the burden of arsenic in the heart mediated by the suppression from As_2_O_3_-induced Nrf2 downregulation (Figure 6). 

In conclusion, the protective role of resveratrol against As_2_O_3_-induced cardiotoxicity is found by the maintenance of redox homeostasis viaNrf2-HO-1 pathway and facilitation of arsenic efflux. Resveratrol has been shown to have antiproliferative effects in various leukemic cell lines [36, 37]. To the best of our knowledge, our findings suggest that coadministration with resveratrol and As_2_O_3_ may be a novel therapeutic strategy for APL. Further investigation is warranted to elucidate another potential signal mechanism by which resveratrol protects As_2_O_3_-induced cardiac injury and another animal model.

## Figures and Tables

**Table 1:** The effect of resveratrol on As_2_O_3_-induced biochemical makers.

	Control	As_2_O_3_	As_2_O_3_ + resveratrol	Resveratrol
AST (U/L)	156.23 ± 56.78	282.69 ± 60.43^a∗∗^	225.78 ± 58.36^b∗^	155.34 ± 53.41
LDH (U/L)	1050.25 ± 370.36	2700.45 ± 380.13^a∗∗^	1680.34 ± 470.68^b∗^	1051.37 ± 360.42
CK (U/L)	336.98 ± 48.04	1463.31 ± 452.16^a∗∗^	597.28 ± 90.54^b∗∗^	335.62 ± 50.26
CK-MB (U/L)	205.83 ± 32.32	812.94 ± 129.85^a∗∗^	407.59 ± 67.40^b∗∗^	204.30 ± 30.02

Values are expressed as mean ± S.E. for eight rats in each group.

*P < 0.05, **P < 0.01; ^a^comparison of control with As_2_O_3_; ^b^comparison of As_2_O_3_ with As_2_O_3_ + resveratrol.
